# Calm after the storm? Similar patterns of genetic variation in a riverine foundation species before and after severe disturbance

**DOI:** 10.1002/ece3.10670

**Published:** 2023-11-01

**Authors:** Magdalene N. Ngeve, Katharina A. M. Engelhardt, Michelle Gray, Maile C. Neel

**Affiliations:** ^1^ Department of Plant Science and Landscape Architecture University of Maryland College Park Maryland USA; ^2^ Department of Entomology University of Maryland College Park Maryland USA; ^3^ Appalachian Laboratory University of Maryland Center for Environmental Science Frostburg Maryland USA

**Keywords:** conservation genetics, disturbance effects, ecological disturbance, genetic bottleneck, genotypic diversity, resilience, submersed aquatic vegetation

## Abstract

In summer 2011, Tropical storms Lee and Irene caused an estimated 90% decline of the submersed aquatic plant *Vallisneria americana* Michx. (Hydrocharitaceae) in the Hudson River of New York (USA). To understand the genetic impact of such large‐scale demographic losses, we compared diversity at 10 microsatellite loci in 135 samples collected from five sites just before the storms with 239 shoots collected from nine sites 4 years after. Although 80% of beds sampled in 2011 lacked *V. americana* in 2015, we found similar genotypic and genetic diversity and effective population sizes in pre‐storm versus post‐storm sites. These similarities suggest that despite local extirpations concentrated at the upstream end of the sampling area, *V. americana* was regionally resistant to genetic losses. Similar geographically based structure among sites in both sampling periods suggested that cryptic local refugia at previously occupied sites facilitated re‐expansion after the storms. However, this apparent resistance to disturbance may lead to a false sense of security. Low effective population sizes and high clonality in both time periods suggest that *V. americana* beds were already small and had high frequency of asexual reproduction before the storms. Dispersal was not sufficient to recolonize more isolated sites that had been extirpated. Chronic low diversity and reliance on asexual reproduction for persistence can be risky when more frequent and intense storms are paired with ongoing anthropogenic stressors. Monitoring genetic diversity along with extent and abundance of *V. americana* will give a more complete picture of long‐term potential for resilience.

## Graphical abstract

Extreme disturbances have potential to reduce genetic diversity through direct loss, and disruption of gene flow patterns and changes in selection pressure. Sampling populations of the submersed aquatic foundation species *Vallisneria americana* before and after massive flood events in the Hudson River, New York, USA, indicated no substantial decline in genetic diversity or change in genetic structure. Recovery from the floods appears to come localized cryptic refugia that expanded through a similar proportion of asexual versus sexual reproduction as prior to the storms.

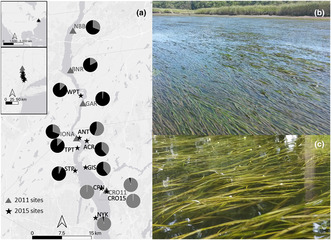

## 1 INTRODUCTION

Extreme, large‐scale weather‐related disturbances such as hurricanes, massive floods, and severe temperatures can cause immediate loss of genetic diversity by abruptly decreasing the number, size, or extent of populations (Banks et al., 2013). Impacts on diversity are expected to be most severe when disturbances exceed historic ranges of variability (Schoennagel et al., 2006), amplify consequences of chronic environmental stressors (e.g., Manent et al., 2020), or are recurrent (Kollars et al., 2021; Schrey et al., 2016). Predictions of increased frequency and magnitude of disturbances (Dale et al., 2001; McPhillips et al., 2018; Michener et al., 1997; Ummenhofer & Meehl, 2017) suggest we could be poised for broad‐scale reductions in genetic diversity in many species. These losses are of concern because genetic diversity confers increased fitness (Amos & Balmford, 2001; Crnokrak & Roff, 1999; Leimu et al., 2006; Weeks et al., 2011; Williams, 2001), enhances growth and productivity (Reynolds et al., 2012; Williams, 2001), and affects ecosystem functioning (Bolnick et al., 2011; Reynolds et al., 2012; Whitlock, 2014) – including resistance to and rapid recovery from disturbances (Ehlers et al., 2008; Hughes & Stachowicz, 2004; Reusch et al., 2005; Unsworth et al., 2015). Although most discussion focuses on dire negative consequences of disturbances, effects on genetic diversity may be neutral to positive (Banks et al., 2013; Foster et al., 2021; Jahnke et al., 2015; Klingbeil et al., 2022; McMahon et al., 2017). Better understanding of when and how genetic diversity is altered by disturbance is needed if we are to know when management interventions are necessary to restore diversity. Such understanding is best gained by comparing diversity from before and after a disturbance (Banks et al., 2013).

Serendipitously, samples of the native submersed aquatic plant, *Vallisneria americana* Michx. (Hydrocharitaceae) were collected (Marsden et al., 2022) just before tropical storms Irene and Lee caused record high floods (Lumia et al., 2014) in the Hudson River of New York (USA), within 2 weeks of each other in late August and early September of 2011. This foundation species is dioecious and forms relatively persistent beds through a combination of sexual and asexual reproduction. When the storms hit, *V. americana* beds were at peak annual biomass; the flowering season was in progress, with fruits mostly still maturing. Shoots would have been starting to produce vegetative overwintering structures called turions. A ~90% reduction in occupancy at long‐term monitoring points in 2012 (Strayer et al., 2014) indicated that scouring and burial by sediment deposition, followed by high turbidity through the end of the growing season, eliminated plants. Occupancy remained low in the subsequent 2 years (Hamberg et al., 2017). When we returned to sample in 2015, many sites that were occupied before the storm remained void of *V. americana*, including four of the five 2011 sampling sites. The magnitude and duration of the demographic losses created immediate concern that the species would no longer be fulfilling its ecosystem functions of providing food and habitat for many species, oxygenating water, and capturing sediments (Findlay et al., 2006; Gurbisz et al., 2017). The losses also set the stage for catastrophic genetic losses. Low genotypic and genetic diversity even before the storms (Marsden et al., 2022) heightened concern that the system had limited capacity to resist or recover from disturbances. On a scale of 0 (all samples are clonal replicates of the same multilocus genotype [MLG]) to 1 (all samples represent unique sexual reproduction events), genotypic diversity (GD) in these sites before the storms ranged from 0.23 to 0.93 (Marsden et al., 2022). And allelic diversity at microsatellite loci was low at all sites, ranging from 2.8 to 3.7 (Marsden et al., 2022).

Loss of individuals and whole populations would have eliminated variation harbored in the extirpated individuals. If the storms reduced population sizes, prevention of or recovery from genetic bottlenecks would require rapid population increases from sexual reproduction; which itself could be limited for several reasons. First, small populations of *V. americana* may not support both sexes (Engelhardt et al., 2014). Sexual reproduction can also be compromised if disturbances reduce plant size because smaller plants have lower frequency of flowering (Engelhardt et al., 2014; Han et al., 2018; Rusterholz et al., 2009). Additionally, if populations are dominated by extensive clones, the sexes may be isolated beyond effective pollination distances, which are on the order of a few meters (Lloyd et al., 2018). Post‐disturbance reestablishment relying on clonal growth allows beds to recover their ecological function quickly. However, beds dominated by replicates of few MLGs will have low effective population sizes (*N*_e_), so genetic diversity will not recover, or may even continue to decline due to genetic drift (Cameron et al., 2011; Soares et al., 2019).

If most shoots at a site are clonal replicates, the only means of increasing genetic diversity naturally is dispersal from other sites. In aquatic plants, most dispersal is accomplished by seeds and vegetative fragments moving among sites via water (Berković et al., 2018; Hall et al., 2006; Lai et al., 2018; Vilas et al., 2017), also see Ngeve et al. (2021). Unfortunately, dispersal may be compromised if disturbance reduces permeability or increases distances among suitable habitat patches beyond a species' dispersal capabilities (Banks et al., 2013; Davies et al., 2016; Orth et al., 2012; Wood et al., 2017). Pre‐storm samples indicated seed dispersal between sites separated by up to ~32 km and vegetative dispersal up to ~17 km (Marsden et al., 2022). In the non‐tidal Potomac River of Maryland (USA), vegetative dispersal has yielded *V. americana* clones that extend 50–160 river km (Lloyd et al., 2011), although dispersal likely occurs in a stepping‐stone fashion across shorter distances.

Comparing amounts and structure of genotypic and genetic variation in *V. americana* before and after massive floods in the Hudson River gave us the rare opportunity to ask which, if any, of these myriad potential effects of disturbance affected genetic variation in this foundation species. Beyond asking if and how demographic losses reduced genetic diversity, we used the genetic data to gain insight into the species' ability to persist via resistance to versus recovery from disturbances. Specifically, we quantified the degree to which beds regrew from individuals that persisted undetected through the storms versus from propagules that dispersed from a few refugial locations. We also determined the degree to which beds expanded through asexual versus sexual reproduction, and if the balance between the modes of reproduction differed from before the storm. In so doing, we assess whether clonality provides a mechanism of resilience by preserving genetic diversity across space and time or if it compromises long‐term resilience and adaptive potential through lowering *N*_e_. Finally, to understand whether the storms changed the spatial distribution of beds in ways that could affect dispersal and future recolonization, we compared the distribution of *V. americana* locations before and after the storms in context of dispersal distances for the species. Through this multi‐faceted inquiry, we test conceptual models of bed formation and persistence and explore implications for resilience of *V. americana* in the Hudson River.

## 2 MATERIALS AND METHODS

### 2.1 Sampling plant material for genetic analyses

**FIGURE 1:**
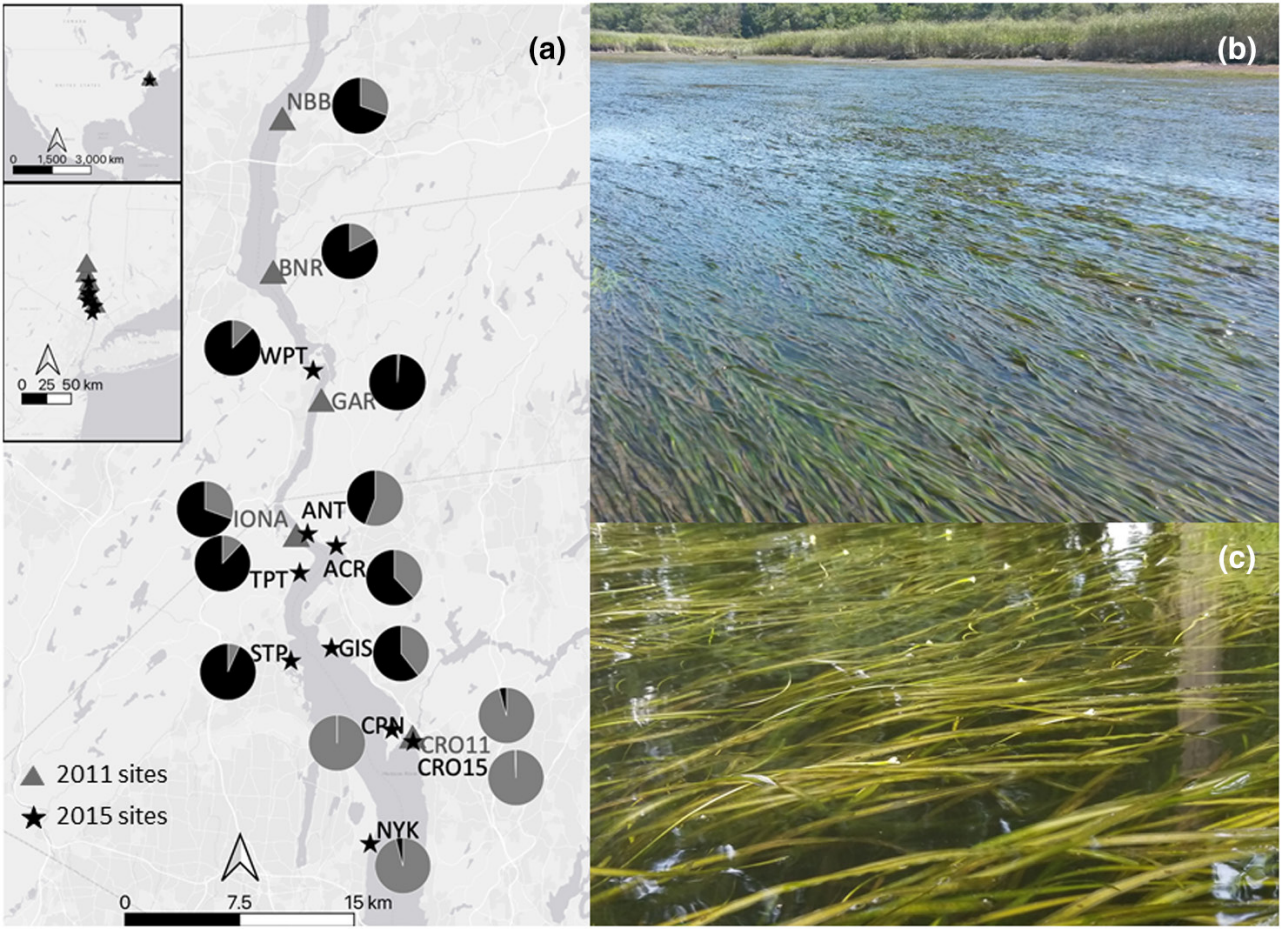
(a) Locations of 14 sampled sites (abbreviations as in Table 1). The proportions of inferred clusters per site (*Q*‐values) from the program Structure (*K* = 2) are represented as pie charts at their respective locations. The structure of genetic variation in samples obtained before and after the storm aligned more with geographical location than with year of sample collection. (b) Dense bed of *Vallisneria americana* in the Hudson River with leaves laying at the water surface at low tide. (c) Female flowers laying among leaves below the water surface.

In summer 2011, 135 *V. americana* samples were collected from 5 sites along a ~50 km stretch of the lower Hudson River Estuary (Figure 1; Table 1; Marsden et al., 2022) just before tropical storms Lee and Irene caused massive flooding. The sites occurred across a salinity gradient and included the most upstream extent of regular salinity influence along the Hudson River. At each site, we attempted to collect 30 shoots spaced 5–15 m apart along two parallel transects, following the sampling protocol of Lloyd et al. (2011). Some sites did not support enough shoots to collect 30 samples, and the distribution of plants within some sites caused variation in distance intervals and transect shape.

**TABLE 1:** Genotypic and genetic diversity estimates for sample sites (codes in parentheses).

Sampling site	*N*	MLG	GD	GD_Shannon_	GD_Simpson_	*A*_t_	*A*_r_	*H*_o_	*H*_e_	*F*_is_	*p* > *F*_is_	*p* < *F*_is_	*N*_e_	*N*_e_ CI
Pre‐storm														
Newburg‐Beacon (NBB)	20	11	0.53	0.44	0.37	3	2.3	0.55	0.40	**−0.34**	1.00	**.0004**	2.3	1.3–8.5
Bannerman Island (BNR)	30	12	0.38	0.20	0.10	3	2.8	0.53	0.49	−0.03	.67	.3296	1.4	0.9–2.2
Garrison (GAR)	27	8	0.27	0.11	0.05	3	2.6	0.57	0.44	−0.24	1.00	.0035	2.1	1.1–10.4
Iona (IONA)	29	17	0.57	0.48	0.40	4	2.9	0.53	0.51	0.02	.37	.6369	27.5	9.7 – inf
Croton (CRO11)	29	27	0.93	0.89	0.82	4	2.8	0.58	0.51	−0.12	1.00	.0042	12.9	7.5–23.8
Mean pre‐storm	27	15	0.54	0.42	0.35	3.4	2.7	0.6	0.47	−0.14			9.2	27
Post‐storm														
West Point (WPT)	2	2	1.00	1.00	1.00	2	na	0.50	0.40	na	na	na	na	na
Antiona (ANT)	33	16	0.47	0.30	0.19	3	2.7	0.60	0.46	**−0.27**	1.00	**.0004**	2	1.4–2.9
Annsville Creek (ACR)	30	24	0.79	0.74	0.67	3	2.8	0.54	0.49	−0.07	.93	.0969	9	4.4–17.2
Turning Point (TPT)	30	13	0.34	0.19	0.09	3	2.6	0.55	0.44	−0.22	1.00	.0015	24.8	6.3 – inf
Georges Island (GIS)	30	7	0.21	0.11	0.08	3	2.9	0.51	0.49	0.04	.38	.7215	26.4	2.3 – inf
Stony Point (STP)	31	18	0.57	0.40	0.27	3	2.4	0.58	0.43	**−0.34**	1.00	**.0004**	4.9	2.2–12.7
Croton Point North (CPN)	23	6	0.23	0.11	0.06	3	2.6	0.70	0.48	−0.38	1.00	.0008	1.1	0.6–2.8
Croton (CRO15)	30	22	0.72	0.61	0.48	4	2.8	0.54	0.50	−0.09	.98	.0373	19.3	9.7–55.9
Nyack (NYK)	30	5	0.14	0.03	0.01	2	2.1	0.52	0.31	**−0.59**	1.00	**.0004**	Inf	Inf–inf
Mean post‐storm	30	14	0.43	0.31	0.23	3.1	2.7	0.6	0.47	−0.19			12.5	

*Note*: Within year, sites are listed from upstream to downstream. *N* = sample size, MLG = number of multilocus genotypes, measures of genotypic diversity (GD = genotypic diversity based on all MLGs, GD_Shannon_ = Shannon's Effective GD, GD_Simpson_ = Simpson's Effective GD), and measures of genetic diversity (*A*_t_ = total number of alleles, *A*_r_ = allelic richness rarefied to 5 diploid individuals, *H*_o_ = observed heterozygosity, *H*_e_ = expected heterozygosity, *F*_is_ = inbreeding coefficient). The proportion of randomization that gave a larger (*p* > *F*_is_, heterozygote deficit) or smaller (*p* < *F*_is_, heterozygote excess) than observed *F*_is_. *S*ignificant *F*_is_ values based on an alpha of 0.05 adjusted for multiple tests (*p* < .00038) are indicated in bold. *N*_e_ = estimates of effective population sizes. *N*_e_ CI = the parametric confidence interval of *N*_e_. All alleles were used in the estimation of *N*_e_. inf = infinite, generated when sample size is too small for estimating of *N*_e_. na = not applicable, because WPT was excluded from mean values and tests because of the small sample size (two individuals). Values from 2011 were previously reported in Marsden et al. (2022). Values in bold indicate sites at which the probability of the observed *F*_is_ value is <0.05.

In summer 2015, we searched for *V. americana* at the 2011 sample sites, at other sites in the same stretch of the river, and ~5 km downstream of the southernmost 2011 site, near the upper salinity limit of the species (Figure 1; Table 1). Most additional sites had been suggested by local managers as being occupied prior to 2011 but had not been surveyed immediately before the storms. We found no *V. americana* at four of the five sites sampled in 2011 despite extensive searches of the surrounding areas to confirm that *V. americana* was really absent. We also surveyed for plants more broadly along routes from our launch points to all sample locations. We were able to collect 239 shoots of *V. americana* from 9 sites, from Nyack (NYK) to West Point (WPT), after the storms (Figure 1). With two exceptions, samples were collected following the Lloyd et al. (2011) protocol used by Marsden et al. (2022) before the storms. The first exception was CPN, where 23 samples were collected by New York Department of Environmental Conservation (DEC) fisheries biologists in fall 2014; they did not sample along parallel transect lines but did adhere to our general distance protocols. The second was WPT, where we collected leaves from the only two plants we found. Because of this small sample size, WPT was included in general summaries but was excluded from all statistical analyses.

### 2.2 Distances among sites and samples

All distance and most other analyses were done using R version 4.3.1 (R Core Team, 2023). GPS coordinates for each sample were converted to Universal Transverse Mercator (UTM) coordinates using the NAD83 datum to allow us to calculate accurate distances. We estimated site centroids as the mean of sample coordinates and used the centroids to calculate pairwise distances among sites. We quantified distances in three ways to assess effects of spatial sampling scale on our results. (1) To understand the extent of sampling, we used the R package riverdist (Tyers, 2022) to calculate distances among sites along the length of the river (termed river distance). To do this, we digitized the midline of the river, added vertexes every meter along that midline using the *line2network* function, and snapped each site centroid to the nearest vertex using *xy2segvert*. We then calculated the distance from each centroid vertex to the mouth of the river (river distance) in kilometers using *riverdistancetofrom*. Finally, we calculated the difference in river distance between each pair of sites and took the maximum difference as the extent of sampled sites. Although it tells us how long a reach of the river was occupied and sampled, river distance does not account for distances across the width of the river. (2) To account for cross‐river separation, we calculated pairwise distances among site centroids and among samples at different sites using either a cost distance that restricted connectivity to water or Euclidean distance, whichever was larger. We restricted movement to water because all pollen and vegetative fragments and most seed dispersal of *V. americana* will be through water. To create the cost surface, we modified a GIS shapefile of the Hudson River Estuary shoreline created by the DEC and downloaded from the New York State GIS Clearinghouse to increase the accuracy of the shoreline by manually digitizing it to match satellite imagery in ArcMap version 10.7.1 (ESRI, 2019). The modified shapefile was converted to a raster with a 10‐m cell size in ArcMap version 10.7.1 (ESRI, 2019). We used that raster to create a transition surface using the *transitionMatrix* function in the R package gdistance using an eight‐neighbor rule and type “c” geocorrection to reflect differences in edge‐to‐edge versus corner‐to‐corner connection (Van Etten, 2017). We assigned water a conductance of 1 and land a conductance of 0 (i.e., it was impassable). We calculated shortest path cost distances among sites through the transition matrix using the *costDistance* function from gdistance. Distances calculated in this manner yield a more accurate representation of dispersal potential between sites than Euclidean distance when the latter cuts across land due to river meanderings and convoluted shorelines. However, raster‐based distance calculations introduce error when samples are close to one another or can be connected with a straight line. The error arises because cost distances are in increments of the transition matrix cell size (10 m in our case) and observations separated by distances less than the cell size will be assigned a distance of 0 because no movement across cells is required. All samples between 10 and 20 m apart will have a distance of 10 m, yielding no sensitivity to local spatial variation. This behavior leads to an underestimate of distance in all *costDistance* calculations; however, its impact declines as the cell size becomes a smaller proportion of the distance between two observations, and as isolation by land becomes more important. (3) To eliminate impacts of this error, we calculated Euclidean distances among all pairs of samples within the same site using the *dist* function in base R. Within sites, samples are not separated from one another by land, so Euclidean distances are far more accurate.

We used the Euclidean distances to quantify spacing among all sample pairs within sites to ensure similar sampling before and after the storms. We used the cost distances to compare distribution and isolation of sites before and after the storms to assess potential changes in connectivity. In absence of any soft barriers, sites separated by shorter distances are expected to be more connected than sites isolated by larger distances. We incorporated differences in the spatial distribution of samples within sites and of sites themselves from the two timepoints in our assessments to understand how the distances might affect genetic patterns and to prevent erroneously attributing differences in distance to disturbance.

### 2.3 DNA extraction, amplification and fragment analyses

We extracted genomic DNA from leaf tissue, using Synergy Plant DNA kits (OPS Diagnostics). We used TopTaq DNA Polymerase Kits (Qiagen Inc.) and species‐specific primers (Burnett et al., 2009) to perform PCR to amplify the same 10 microsatellite loci using the same PCR conditions as Marsden et al. (2022). Initial denaturation of 95°C for 15 min was followed by 34 cycles of 30 s denaturation, 90 s of annealing at a temperature of 57°C and elongation of 80 s at 72°C. These cycles were followed by a final elongation time of 30 min at 60°C and cooling to 4°C for 1 min. PCR products were labeled with fluorescent tagged forward primers and separated and measured on the University of Maryland College Park Applied Biosystems 3730xl DNA Analyzer with GeneScan™‐500 using a 500 LIZ™ size standard (Applied Biosystems/ThermoFisher Scientific). Peak data were analyzed using GeneMapper v3.7 (Applied Biosystems) and all allele calls were inspected and made consistent with previously analyzed data following standards and decision rules of Lloyd et al. (2011) and Marsden et al. (2022). Ambiguous calls were re‐genotyped and if the call remained ambiguous after 3–4 attempts, the alleles were coded as missing.

### 2.4 Data quality assessment

We first quantified missing data using a summary of an adegenet (Jombart, 2008) genind object containing all samples. We then asked if there were deviations from Hardy–Weinberg Equilibrium (HWE) of unique MLGs overall and within sites within each sample period using *hw.test* function from the package pegas (Paradis, 2010). Finally, we tested for linkage disequilibrium among loci using the *pair. ia* function from poppr (Kamvar et al., 2014), and for null alleles using *null. all* from PopGenReport (Adamack & Gruber, 2014) using both methods offered (Brookfield, 1996; Chakraborty et al., 1994).

### 2.5 Identifying multilocus genotypes

Shoots collected in 2011 were assigned to multilocus genotypes (MLGs) based on complete allele matches at all loci using Genodive v2.0b17 (Meirmans & Van Tienderen, 2004). Assignments were manually checked to retain only those that were unique MLGs despite missing loci (Marsden et al., 2022). The post‐storm MLG assignments were made using the *mll* function in poppr, following the same decision rules used for 2011 samples. To determine if new collections were the same MLG as 2011 collections, we assigned samples from all years to MLGs and filtered for MLGs that included samples from both time periods. In such cases the new samples were assigned the original MLG code. Each new MLG was assigned a unique code.

Using all samples, we assessed probabilities of multiple chance encounters of the same MLG from sexual reproduction following Parks and Werth (1993) and Arnaud‐Haond et al. (2007; with 2008 errata correction, 2010) as implemented by poppr. For MLGs occurring more than once, we calculated *p_sex_* using the *multiple* method to get the probability of seeing the MLG the number of times it was observed. We calculated *p_sex_* separately for each site and for all sites combined. We considered each additional occurrence of an MLG beyond the first to be a member of the same clone if *p_sex_* was <0.01.

### 2.6 Comparing genotypic diversity

Overall and within‐site numbers of MLGs, as well as Shannon's and Gini‐Simpson indices were quantified using the poppr *mlg* function. We calculated effective numbers of MLGs by taking the exponent of the Shannon index and the reciprocal of the Gini‐Simpson index (Jost, 2007). Effective numbers give the number of equally abundant MLGs that would yield the observed index value. If all samples yield distinct MLGs, they are equally abundant and the three measures are the same. As abundances of MLGs become increasingly uneven, effective numbers diverge in a predictable rank order with richness > effective Shannon's > effective Gini‐Simpson's. This rank emerges because richness emphasizes rare entities, Shannon's index treats all entities in accordance with their abundance, and the Gini‐Simpson index emphasizes common entities. Because effective numbers respond monotonically to changes in genotypic diversity (Jost, 2007) they allow us to compare dominance versus evenness of MLGs across sites and time periods, which in turn reflect amounts and nature of clonal versus sexual reproduction. To limit the influence of sample size on estimates of MLG diversity, we calculated overall and within‐site genotypic diversity (GD) as (*G* − 1)/(*N* − 1), where *G* is the number of unique MLGs in *N* genotyped shoots (Arnaud‐Haond et al., 2007). GD equals 1 when all samples are different MLGs and is 0 when they are the same MLG (i.e., the same clonal individual). We used effective numbers of genotypes to calculate GD_Shannon_ and GD_Simpson_.

To understand how the floods altered dominance of MLGs, we compared GD, GD_Shannon_ and GD_Simpson_ before versus after the storms calculated once based on all samples from each year and then based on samples within each site. Comparisons based on all samples combined yielded one value of each measure per sampling time, so no statistical tests were possible. To quantify the probability of seeing the observed differences in site‐level GD in the two time periods by chance, we permuted values across sites 10,000 times using the *permutations* function in the modelr package version 0.1.11 (Wickham, 2023). We calculated the absolute value of the difference between the mean in each time period for each permutation and the observed data. The proportion of permuted values that were larger than the observed was recorded as the probability of the observed value occurring by chance. Non‐significant values are reported to the second decimal place.

To gain more insight into how clonal reproduction was affected by the storms and to assess its role in recovery, we further explored distribution and dominance of MLGs by counting the number of MLGs observed only once versus the number observed multiple times during each time period. For each MLG detected more than once, we counted how many times it was sampled and measured the maximum distance between samples in each time period. We made note of MLGs found in both time periods.

We tested for differences in the proportions of single versus multi‐shoot MLGs in the two time periods overall and within sites. To understand changes in number of shoots per MLG, we examined the number of shoots of all individuals and the number of shoots of only multi‐shoot individuals. To understand if clonal growth differed before versus after the storms, we tested the probability of seeing the observed differences between time periods using permutation tests. Site‐level comparisons were done as described previously. When asking if MLGs differed, attributes were permuted across MLGs. As in our other permutation tests, we compared the observed data against the permuted distribution of absolute differences in mean values in each time period.

To understand effects of the storms on MLGs known to survive through them, we compared the number of shoots and maximum distance between them in both time periods combined, with those same values only before and only after the storms. We took the difference between the maximum values for each MLG across both time periods versus only after the storm to represent change due to the storm.

### 2.7 Genetic diversity

To compare genetic diversity before and after the storms, we calculated overall and within‐site genetic diversity statistics using only one representative for each MLG within each site. For all samples combined, we summarized the number of alleles found, highlighting the number found exclusively in each time period versus the number of alleles shared between the two time points using the private alleles function in GenAlEx (Peakall & Smouse, 2012). We also generated allele accumulation curves for each time period using the *specaccum* function in the vegan package with 10,000 permutations (Oksanen et al., 2022).

For all samples within each time period and within each site, we calculated number of total alleles per locus (*A*_t_), observed heterozygosity (*H*_o_), expected heterozygosity (*H*_e_) using GenAlEx and calculated the inbreeding coefficient (*F*_is_) using FSTAT (Goudet, 1995, 2003). To account for differences in sample size in the estimation of the number of alleles, we also calculated allelic richness (*A*_r_), rarefied to the smallest number of MLGs at sites excluding WPT (5 diploid individuals) using FSTAT. Measures of genetic diversity other than *A*_r_ were not rarefied. We compared pre‐storm versus post‐storm samples using the group comparison method in FSTAT. Probability of the observed differences occurring by chance was assessed as the proportion of 10,000 permutations with a larger value of the test statistic than observed value (Goudet, 2003). We tested for differences site‐level genetic diversity (excluding WPT) before versus after the storms using the same permutation framework for other site‐level tests described previously.

We knew from prior analysis (Marsden et al., 2022) that three of the five 2011 sites had evidence of population genetic bottlenecks. To investigate if bottlenecks were indicated after the storms, we tested all 13 sites (i.e., excluding WPT) for heterozygote excess and shifts in allele frequency distribution using the program Bottleneck v1.2.02 (Piry et al., 1999). We used the two‐phase mutation (TPM) model because it is preferred for SSR markers (Di Rienzo et al., 1994) in that it gives results that are intermediate between an infinite allele model and a stepwise mutation model (SMM). The proportion of the SMM in the TPM was set at 0.000, and the TPM's variance of geometric distribution was 0.36, as recommended for microsatellites. To test for evidence of a recent bottleneck, we ran 10,000 iterations and used a one‐tailed Wilcoxon sign‐rank test with *p* < .0038 to allow for multiple comparisons to determine if heterozygosity exceeded what is expected at mutation‐drift equilibrium.

We used the program *N*_e_ Estimator Version 2 (Do et al., 2014) to calculate *N*_e_ of all sites using the bias‐corrected version of the linkage disequilibrium (LD) method (Jones et al., 2016; Waples, 2006; Waples & Do, 2010). The LD method is preferred when using microsatellite data because it outperforms the temporal method except when samples are taken several generations apart (Waples & Do, 2010). As with all linkage disequilibrium methods, LD underestimates *N*_e_ when individuals are distributed continuously rather than in discrete populations (Neel et al., 2013). However, we expect the bias to be the same in both time periods such that we can use the estimates to detect changes. Following the program manual, we chose an allele frequency critical value (*P*Crit) of 0+, to include all alleles in the analyses (also see Do et al., 2014). We tested for significant differences in *N*_e_ estimates between the time periods using the same permutation test method described previously.

### 2.8 Population genetic differentiation

We used four methods to assess changes in the structure of genetic variation among sites sampled before versus after the storms. First, we used GenAlEx to estimate pairwise actual allelic differentiation (*D*_est_) among all site pairs within each of the two temporal groups with 9999 permutations. Second, we carried out three analysis of molecular variance tests based on allele identity (AMOVA‐*F*_st_). To evaluate variation accounted for by year of sampling, we implemented a hierarchical AMOVA‐*F*_st_ that considered samples nested in sites in the time from which samples were collected (i.e., pre‐storm versus post‐storm). The other two AMOVA‐*F*_st_ analyses used samples within each time period separately to determine if the sources of molecular variation among samples differed between the two time periods. Missing data were interpolated and significance of all AMOVA analyses was assessed using 9999 permutations.

Third, principal coordinate analysis (PCoA) was done in GenAlEx using Nei's genetic distance among sample sites. We asked if similarity in ordination scores was more related to the time period of sampling or the spatial locations of sample sites. If sample sites were eliminated by the storms and recolonized by few individuals from a small pool of surviving founders, we would expect sites from 2015 to be more similar to each other than were the 2011 sites and to represent a subset of variation seen in 2011. By contrast, if proximity in ordination space is more closely aligned with geographic proximity, either persistence through the storms and re‐expansion, or recolonization from multiple localized sources that survived the floods would be more parsimonious. We also calculated the percent of difference in the extents of the first three ordination gradients that were occupied when all sites were include versus without the four extirpated sites to understand changes in genetic variation in genetic distance represented by PCoA scores after the storms.

Fourth, we used Bayesian clustering analysis in Structure (Pritchard et al., 2000) to assign all samples combined into inferred genetic clusters and to determine whether inferred clusters reflected spatial proximity of the sampling locations or year of collection. We also examined 2011 and 2015 separately to determine if population structure among sample sites was altered by the 2011 storms. We assumed a non‐admixture model and tested *K* values from 1 to 10, with 10 iterations for each *K*. The Markov Chain Monte Carlo (MCMC) repetition was set at 1 × 10^6^ after a burnin of 100,000. The program assumes minimal deviations from HWE and linkage equilibrium. STRUCTURE HARVESTER online (Earl & vonHoldt, 2012) was used to visually determine the best *K* value using the Evanno method of the highest Δ*K* value. For each site, the proportion of inferred cluster for each site (the *Q* score) was visualized using pie charts.

## 3 RESULTS

### 3.1 Distances among sites and samples

**FIGURE 2:**
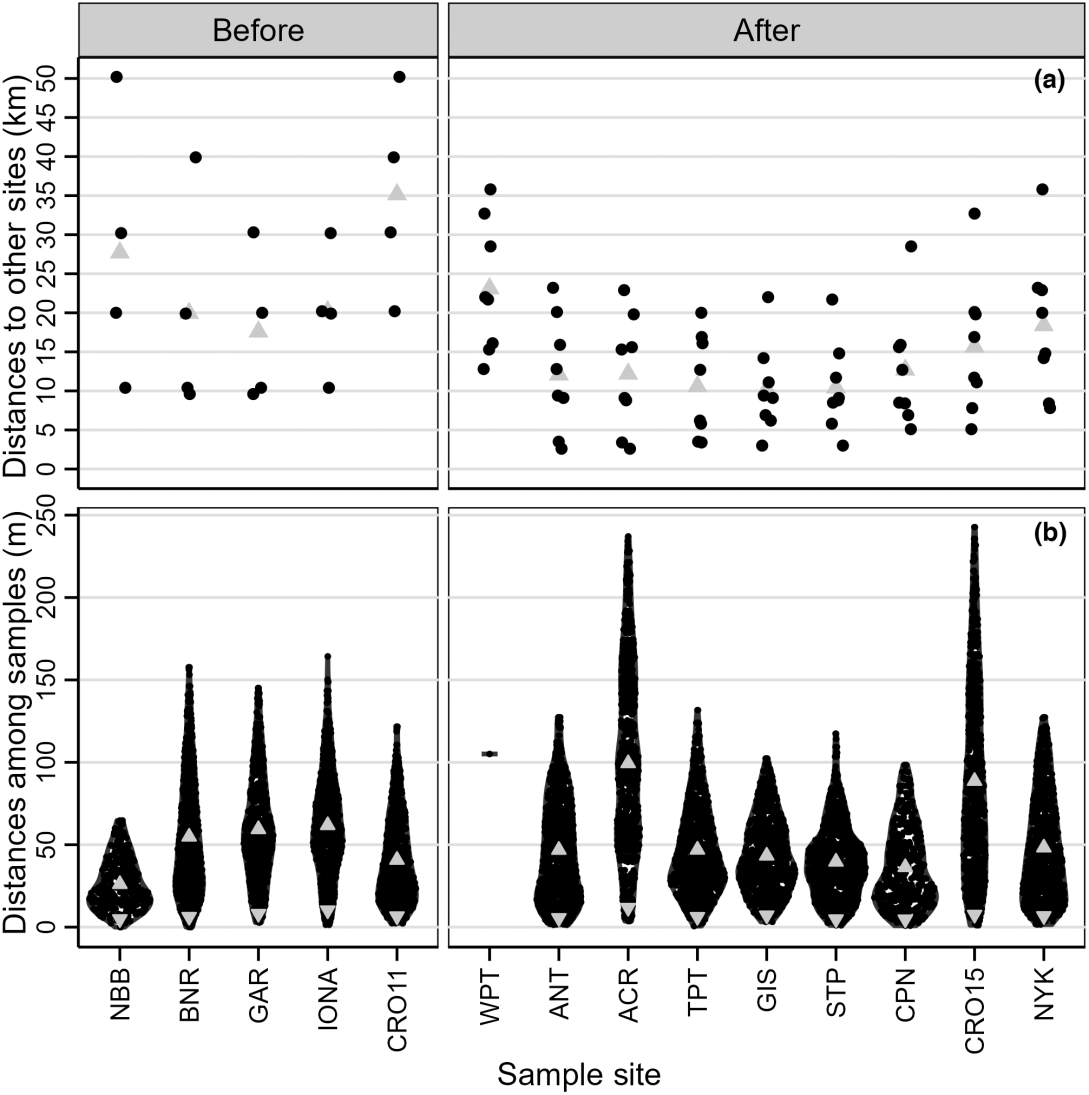
(a) Pairwise distance (km) through water from each site to all other sites sampled in the same time period (black dots) with the mean distance to all other sites (upward facing gray triangles). Within each time, period sites are ordered upstream to downstream. (b) Pairwise Euclidean distances (m) among all samples within sites. Sites are arranged from upstream to downstream within sample year. Upward facing triangles represent mean distances among all sample pairs within sites. Downward facing triangles represent mean distances between each sample and its closest neighbor sample.

Sites sampled in 2011 spanned ~50.2 river km in the lower Hudson River Estuary (Figure 1) and each site was 9.6–20.2 km through water from its nearest neighbor ($\bar{x}$ = 12.0 km; Figure 2a). In 2015, we attempted to collect samples from the stretch of the river that encompassed all 2011 sites and further downstream. The four 2011 sites that had been extirpated were all in the most upstream part of our sampling area (Figure 1), reducing the river distance over which we collected samples to 35.8 km. On average, sample sites were 5.0 km through water from the nearest‐neighbor site (range = 2.6–12.8 km; Figure 2a). If we exclude WPT to focus only on sites supporting beds, the occupied river distance was only 23.2 km and average nearest‐neighbor distance was 4.1 km (range = 2.6–7.8 km). Thus, the storms reduced the extent of the lower Hudson River over which we detected *V. americana* in 2015 (Figure 1), and the sites we found were closer to one another (Figure 2a) than those sampled in 2011.

Within sites, samples were on average 7.2 m away from the closest neighboring sample in 2011 and 6.8 m away in 2015. The range of distances among samples within sites was similar before and after the storms except at ACR and CRO15 (Figure 2b), where low plant density necessitated collecting over larger areas. At these sites, the most distant samples were up to ~242 m apart, which was ~100 m further apart than at other sites. ACR had the highest average distance among nearest‐neighbor samples (11.8 m) and among all sample pairs (99 m; Figure 2b). At CRO15, the overall average distance was second highest of all sites at 89 m, but the average distance among neighboring samples (7.6 m) was similar to other sites. The expected effect of larger distances among samples is detecting more unique MLGs than would be yielded by samples collected closer together. We incorporated differences in distance among (Figure 2a) and within (Figure 2b) sites in our interpretation of diversity patterns.

### 3.2 Data quality assessment

In 2011, 0.44% of data for all samples was missing due to non‐amplification or ambiguous allele calls. In 2015 this percentage was even lower, with 0.04% of data missing. Based on exact tests on unique MLGs from all sites combined, only one locus was out of HWE in each time period. When sample sites were examined separately, Monte Carlo sampling indicated three sites had loci that appeared to deviate from HWE based on *p* < .05 (four loci at BNR and one each at CRO11 and GAR). In 2015 deviation from HWE was indicated for one locus at ANT and two loci at STP. As is common in small populations with low diversity, linkage disequilibrium was detected in both time periods with index of association values of 0.68 in 2011 and 0.44 after the storms. The highest index of association within sites in 2011 was 0.31 and in 2015 the highest was 0.19. We saw no evidence of null alleles at any locus based on either method implemented in PopGenReport.

### 3.3 Comparing genotypic diversity

The 135 pre‐storm samples (Marsden et al., 2022) were assigned to 73 MLGs (GD = 0.54) compared to 110 MLGs for 239 post‐storm samples (GD = 0.46). Values of *p_sex_* for MLGs that occurred multiple times were always ≪0.01, so replicate occurrences of MLGs were considered to be members of the same clonal lineage. Five MLGs were sampled in both time periods, yielding an overall total of 178 MLGs from 374 samples (overall GD = 0.47). GD_Shannon_ was 0.34 before the storms, 0.23 after, and 0.25 in both time periods combined. GD_Simpson_ for the same comparisons were 0.18, 0.11, and 0.12, respectively. These GD values indicate that overall, clonal reproduction was common before the storms and even more common after the storms. The differences between GD and GD_Simpson_ and GD_Shannon_ indicated highly uneven abundances of MLGS that became more uneven after the storms.

**FIGURE 3:**
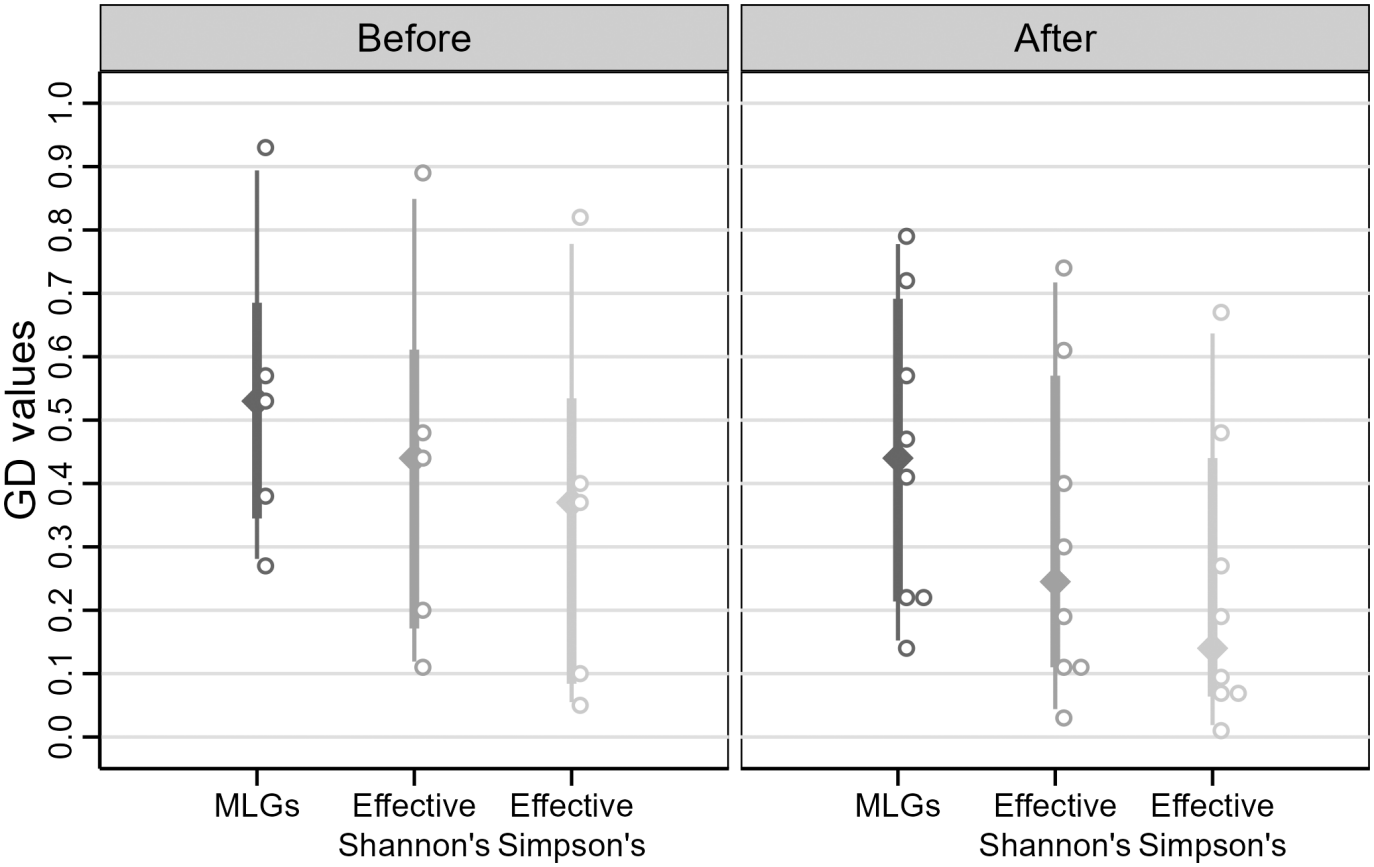
Overall genotypic diversity (GD) for both time periods calculated using three indices: all multilocus genotypes (MLG's), effective Shannon's and effective Simpson's indices. Comparing the three measures shows that abundances of multilocus genotypes were uneven in both time periods and became slightly more uneven after the storms.

The mean number of MLGs within sites before the storms was 15 (mean GD = 0.54) compared to 14 after (mean GD = 0.44) and these values did not differ (*p* = .52). At the one site we were able to sample before and after the storms (CRO11 and CRO15), GD decreased from 0.93 to 0.72, but still had the third highest site‐level GD (Table 1). Comparison of GD, GD_Shannon_, and GD_Simpson_ revealed uneven abundance among MLGs in both time periods (Figure 3). As with GD based on richness, within‐site GD_Shannon_ or GD_Simpson_ before versus after the storms did not differ from a randomly permuted distribution (*p* = .49 and *p* = .46, respectively).

**FIGURE 4:**
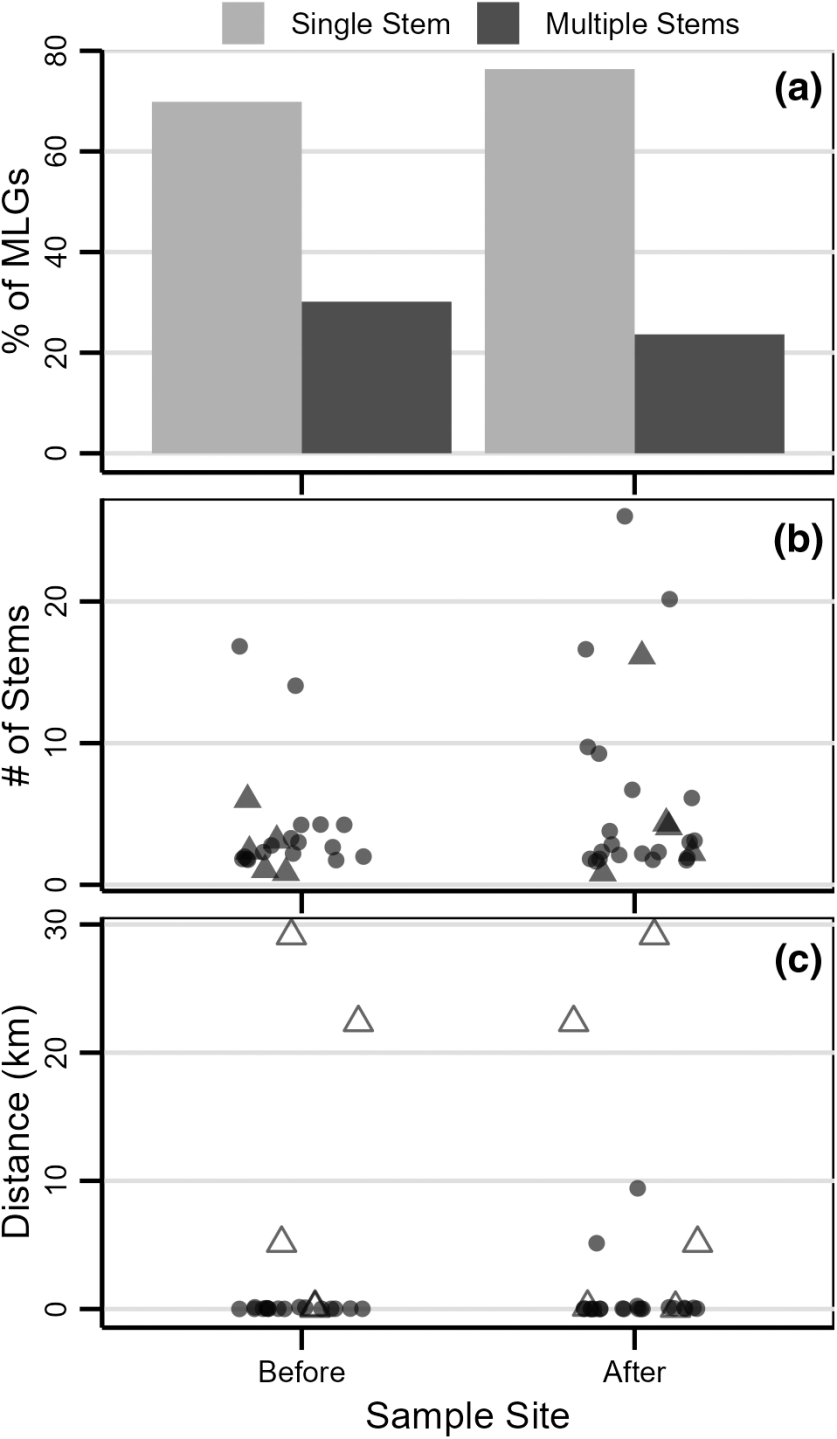
(a) Percent of multilocus genotypes at each site that were sampled once versus multiple times. (b) Number of stems for each multi‐stemmed individual. Open triangles indicate MLGs found in both time periods. (c) Distances between instances of the same MLG showing MLGs shared within and across time periods.

**FIGURE 5:**
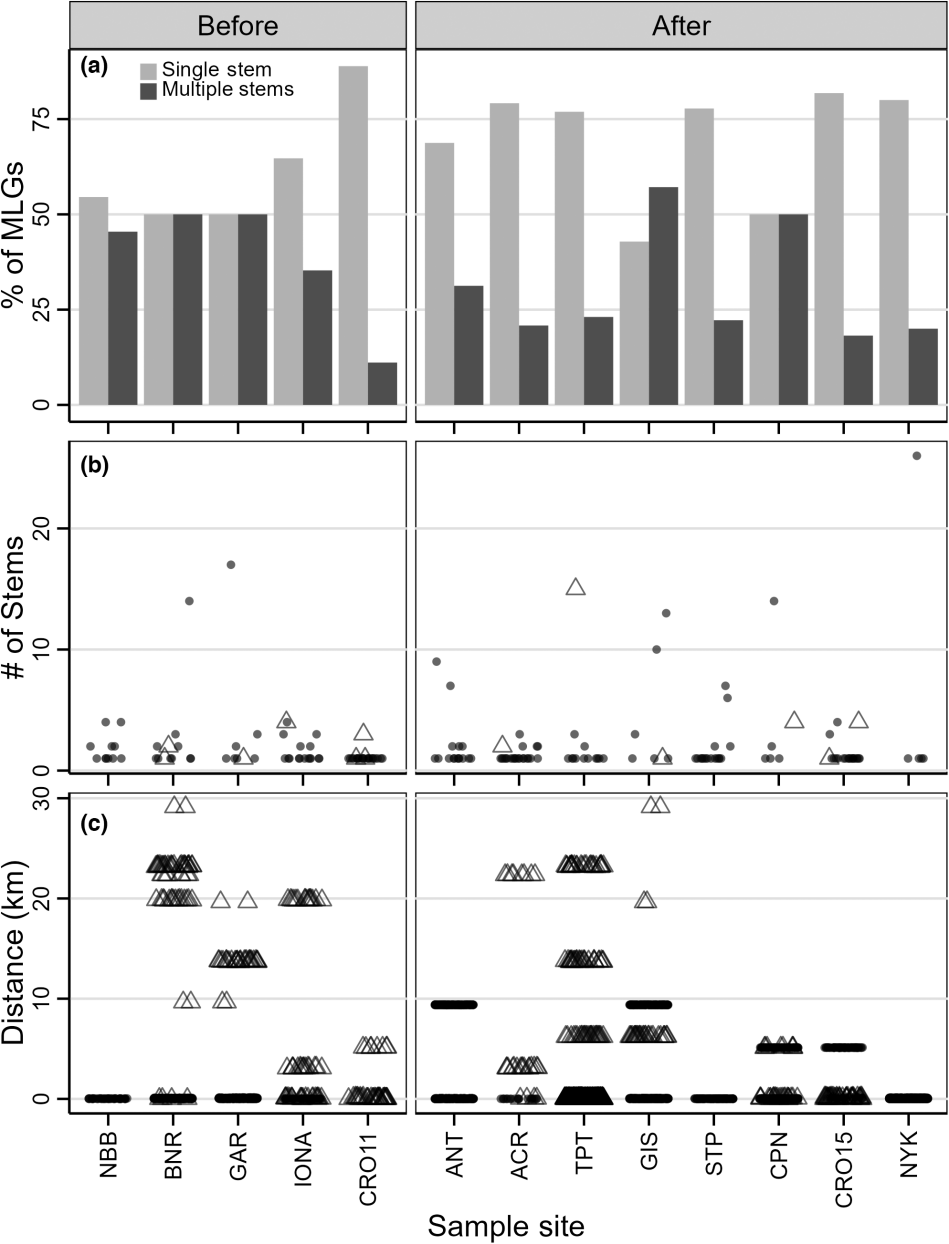
(a) Percent of MLGs with single versus multiple stems within sites. (b) Number of stems within each site for each multi‐stemmed individual. (c) Distances between instances of the same MLG, showing MLGs shared within and across sites and within and across time periods. Open triangles denote MLGs found in both time periods.

We also saw no increase in number of MLGs with multiple shoots. Overall, we detected 22 multi‐shoot MLGs (30.1% of the 73) before the storms and 26 MLGs (23.6% of the 110) after (*p* = .39; Figure 4a). These multi‐shoot individuals accounted for 62% of the shoots sampled before the storms and 64% after. Within sites, an average of 38.4% MLGs sampled before the storms had multiple shoots compared with 30.3% of MLGs within sites sampled after (Figure 5a) and these values did not differ (*p* = .37).

Additionally, we found no difference in overall number of shoots per MLG before ($\bar{x}$ = 1.8 shoots) versus after the storms ($\bar{x}$ = 2.2 shoots; *p* = .51), mean number of shoots per MLG within sites ($\bar{x}$ = 2.1 shoots before and $\bar{x}$ = 2.8 after; *p* = .39), or maximum number of shoots per MLG within sites ($\bar{x}$ = 8.4 shoots before and $\bar{x}$ = 11.4 after; *p* = .50). We also found no differences in numbers of shoots when only multi‐shoot MLGs were considered, either when we looked at mean values overall ($\bar{x}$ = 3.8 shoots before and $\bar{x}$ = 6.0 shoots after; *p* = .20; Figure 4b) or within sites ($\bar{x}$ = 4.1 before and $\bar{x}$ = 7.8 after; *p* = .38; Figure 5b). The spatial extent of these multi‐shoot MLGs did not differ, with the average maximum extent of 3.48 km before and 2.62 after (*p* = .63; Figure 4c).

Most multi‐stemmed MLGs were restricted to one site in one time period (17 before and 19 after the storms). Shoots of these MLGs were sampled ≤145 m apart before the storms and ≤233 m after, with an average maximum distance of ~42 m in both time periods. Five MLGs were detected at multiple sites at distances ranging from 5.1 to 29.1 km through water (Figure 5c). Two of these MLGs were each shared by two post‐storm sites: GIS and ANT (9.4 km apart) and CRO15 and CPN (5.1 km apart). The remaining three multi‐stemmed MLGs at different sites were found in different time periods – one MLG sampled at CRO11 was also found 5.1 km upstream at CPN in 2014; an MLG shared by BNR and IONA (separated by 19.9 km) in 2011 was also found at ACR in 2015 (~3.2 km downstream from IONA). Finally, an MLG sampled at BNR and GAR (separated by 9.6 km) in 2011 was detected in 2015 at GIS and TPT (separated by 6.2 km). The 2015 sites were 13.7–29.1 km downstream from the 2011 sites. We also found two MLGs in both time periods at CRO11 and CRO15.

The five MLGs found in both time periods were detected 1–6 ($\bar{x}$ = 2.6) times before the storms and 1–16 ($\bar{x}$ = 5.4) times after. If the four 2011 sites had not been extirpated and abundances remained similar across time, the number of shoots per MLG combined across years would have ranged from 2 to 18 ($\bar{x}$ = 8). Total extents of the 5 MLGs ranged from 63 m to 29.1 km ($\bar{x}$ = 11.4 km). Post‐storm extents reached a maximum of 6.2 km and averaged 1.6 km.

### 3.4 Comparing genetic diversity

**FIGURE 6:**
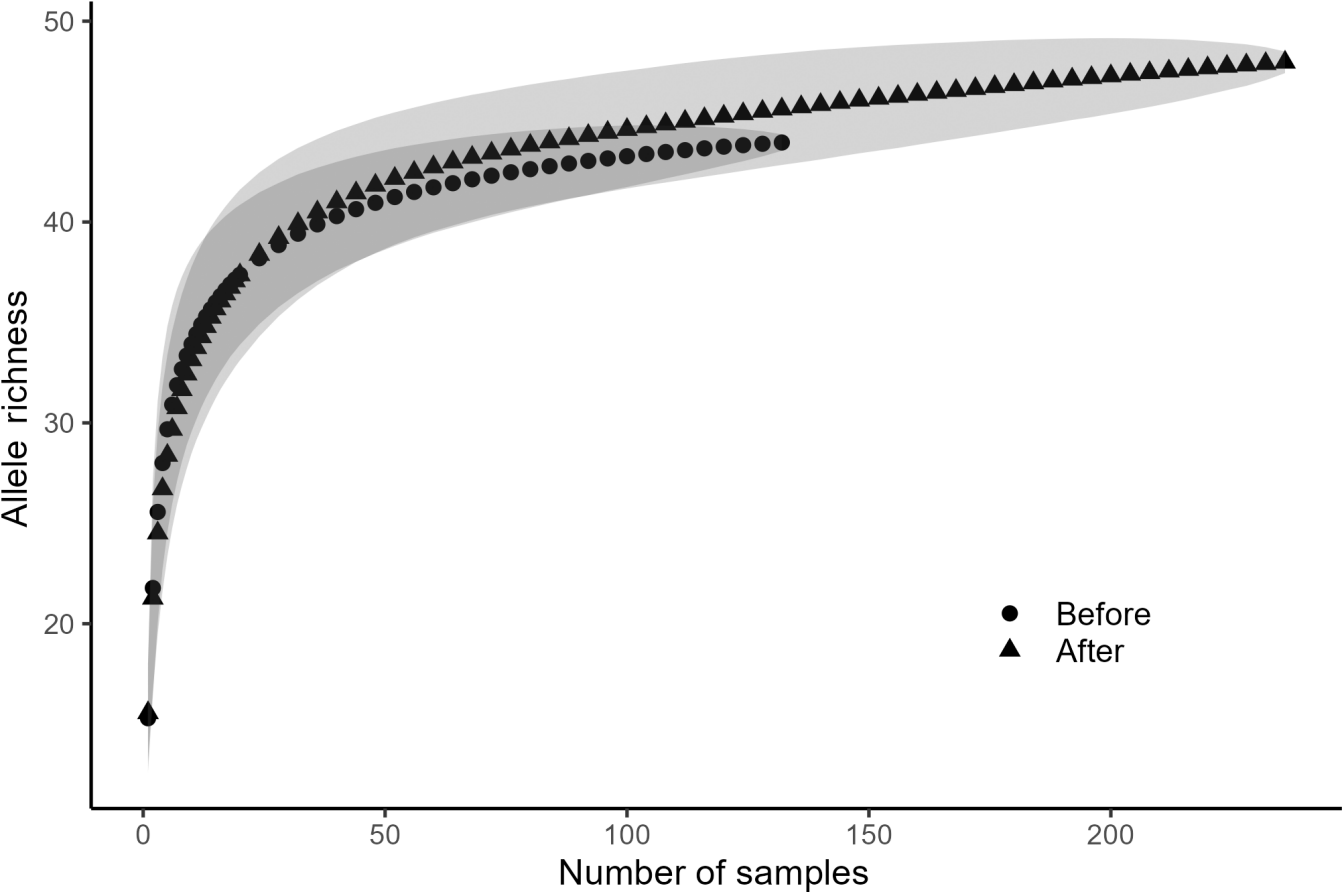
Allele accumulation curves for samples collected before versus after the storms generated using the rarefaction option in the specaccum function in the R package vegan. Error bars are ±2 SD based on 10,000 permutations. Numbers of samples were thinned to improve visualization. Every number of samples between 1 and 20 is shown; between 20 and the maximum number of samples for each year, only every fourth sample is shown.

We found 52 alleles in all samples combined; 44 were found before the storms and 48 were found after. Four of the alleles found before the storms were not observed after, and 8 alleles found post‐storm were not observed pre‐storm. Alleles found exclusively either pre‐ or post‐storm ranged in frequency from 0.007 to 0.054, whereas those shared among time periods ranged in frequency between 0.007 and 1.000 before the storms, and 0.004–0.973 after. Allele accumulation curves were similar in both time periods with confidence intervals overlapping throughout (Figure 6). Based on group‐level comparison, none of the measures of genetic diversity (*A*_r_, *H*_o_, *H*_s_ [~*H*_e_], or *F*_is_) differed before versus after the storms (Table 2).

**TABLE 2:** Genetic diversity (rarefied allelic richness = *A*_r_, observed heterozygosity = *H*_o_, gene diversity = *H*_s_, inbreeding coefficient = *F*_is_) and differentiation (based on allele frequencies = *F*_st_) did not differ between pre‐storm and post‐storm sample groups (*p* values shown below).

Sample group	*A*_r_	*H*_o_	*H*_s_	*F*_is_	*F*_st_
Pre‐storm (2011)	2.686	0.551	0.498	−0.107	0.125
Post‐storm (2015)	2.609	0.560	0.473	−0.183	0.122
*p* Values	.5952	.6724	.3237	.3474	.948

Site‐level measures of genetic diversity excluding WPT were also similar before and after the storms (Table 1). On average, sites sampled before the storms supported 3.4 alleles per locus (*A*_t_) and sites sampled after supported 3.1 (*p* = .33). Allelic richness (*A*_r_), rarefied to the smallest number of unique MLGs, ranged from 2.3 to 2.9 before the storms and from 2.1 to 2.9 after (*p* = .66). Allelic richness at Croton remained the same before and after the storms (*A*_t_ = 4 and *A*_r_ = 2.8). *H*_o_ ranged from 0.516 to 0.580 for pre‐storm sites, and from 0.510 to 0.697 for post‐storm sites (*p* = .71). *H*_e_ ranged from 0.395 to 0.513 pre‐storm and from 0.314 to 0.495 post‐storm (*p* = .60). Negative *F*_is_ in 11 out of 13 sites indicated no inbreeding; in fact, there was marginally significant heterozygote excess at 4 sites (NBB, ANT, STP, and NYK; Table 1); and *F*_is_ levels were similar at both time points (*p* = .37).

Significant recent bottlenecks were indicated in six sites based on shifts from the allele frequency distribution that is expected under mutation‐drift equilibrium (Table 3). Three of those sites were sampled before the storms (BNR, IONA, GAR) and three after (ACR, GIS, and NYK; Table 3). No sites had significant heterozygote excess when the significance of the Wilcoxon sign‐rank test was adjusted for multiple tests (*p* < .0038). If alpha was not adjusted (i.e., *p* < .05), significant bottlenecks would also have been suggested at CRO11 before the storms and ANT and CPN after (Table 3).

**TABLE 3:** Results of BOTTLENECK (Piry et al., 1999) analysis with a two‐phase mutation (TPM) model, where we set the proportion of the stepwise mutation model (SMM) in the TPM to 0.000 and a variance of the geometric distribution for the TPM to 0.36, as recommended for microsatellites by the program authors.

Population	Mean *N*	Mean *K*	Probability of heterozygote excess	Allele frequency distribution
Pre‐storm (2011)				
NBB	22.	2.8	.326172	Normal L‐shaped
BNR	23.4	3.4	.018555	**Shifted**
GAR	16.	2.9	.125000	**Shifted**
IONA	33.8	3.6	.018555	**Shifted**
CRO11	53.6	4.0	.006836	Normal L‐shaped
Post‐storm (2015)				
ANT	32	3.3	.027344	Normal L‐shaped
ACR	48	3.4	.003906	**Shifted**
TPT	26	3.2	.285156	Normal L‐shaped
GIS	14	3.1	.013672	**Shifted**
STP	36	2.9	.064453	Normal L‐shaped
CPN	11.8	3.6	.013672	Normal L‐shaped
CRO15	44	3.7	.096680	Normal L‐shaped
NYK	10	2.1	.218750	**Shifted**

*Note*: Sites with evidence of bottlenecks are in bold, as detected by either significant probabilities from a one‐tailed Wilcoxon sign‐rank test or shifted allele frequency modes. *N* = number of genotyped shoots; *K* = mean partition value. Based on the adjusted alpha of .00038 for multiple comparisons, none of the sites were significant for recent genetic bottleneck. WPT was not included in the analyses, because we found only two individuals at the site.

Contemporary *N*_e_ was very low for all sites in both time periods, ranging from 1.1 at CPN to 27.5 at IONA (Table 1). *N*_e_ could not be estimated at post‐storm sites NYK and WPT due to too few unique MLGs or samples, respectively. There was no difference in estimated *N*_e_ in pre‐storm versus post‐storm sites (*p* = .92).

### 3.5 Genetic differentiation and connectivity among sites

Pairwise *D*_est_ among sites before the storms was significantly different from 0 except between BNR and GAR (Table 4a) and significant values ranged from 0.037 (between IONA and BNR) to 0.265 (between GAR and CRO11). After the storms, CRO15 did not differ from CPN, and GIS did not differ from ANT, ACR, TPT, or CPN (Table 4b). In the remaining pairs of post‐storm sites, *D*_est_ ranged from 0.08 (between ANT and TPT) to 0.278 (between STP and NYK). Hierarchical analyses of molecular variance of sites nested in years based on allele frequencies (AMOVA‐*F*_st_) indicated significant variation among sites (*F*_st_ = 0.113, *p* = .000), even among sites sampled within the same year (*F*_sg_ = 0.122, *p* = .000), but no variance was accounted for by year. Group comparison also showed that *F*_st_ among 2011 sites did not differ from 2015 sites (*p* = .9198; Table 2). When pre‐ and post‐storm sites were analyzed separately, AMOVAs both indicated significant (*p* = .001) substructure among sites before (*F*_st_ = 0.124) and after (*F*_st_ = 0.122) the storms.

**TABLE 4:** Pairwise *D*_est_ of pre‐storm (2011) populations (a) and post‐storm sites (b).

(a) Pre‐storm sites	NBB	BNR	GAR	IONA	CRO11
NBB	0	0	0	0	0
BNR	0.084	0	**0.124**	0.041	0
GAR	0.155	**0.028**	0	0.003	0
IONA	0.132	0.037	0.088	0	0
CRO11	0.182	0.213	0.265	0.102	0

(b) Post‐storm sites	ANT	ACR	TPT	GIS	STP	CPN	CRO15	NYK
ANT	0	0.001	0.001	**0.32**	0	0.001	0	0.001
ACR	0.054	0	0.002	**0.05**	0	0	0	0
TPT	0.08	0.048	0	**0.271**	0	0	0	0
GIS	**0.004**	**0.033**	**0.009**	0	0	0.024	0	0.009
STP	0.122	0.13	0.086	0.081	0	0	0	0
CPN	0.098	0.155	0.205	0.088	0.213	0	**0.076**	0.002
CRO15	0.146	0.181	0.193	0.12	0.146	**0.03**	0	0
NYK	0.136	0.199	0.256	0.116	0.278	0.144	0.192	0

*Note*: *D*_est_ values are below the diagonal and *p* values based on 9999 permutations are above the diagonal, *p* values ≥ .05 are in bold.

**FIGURE 7:**
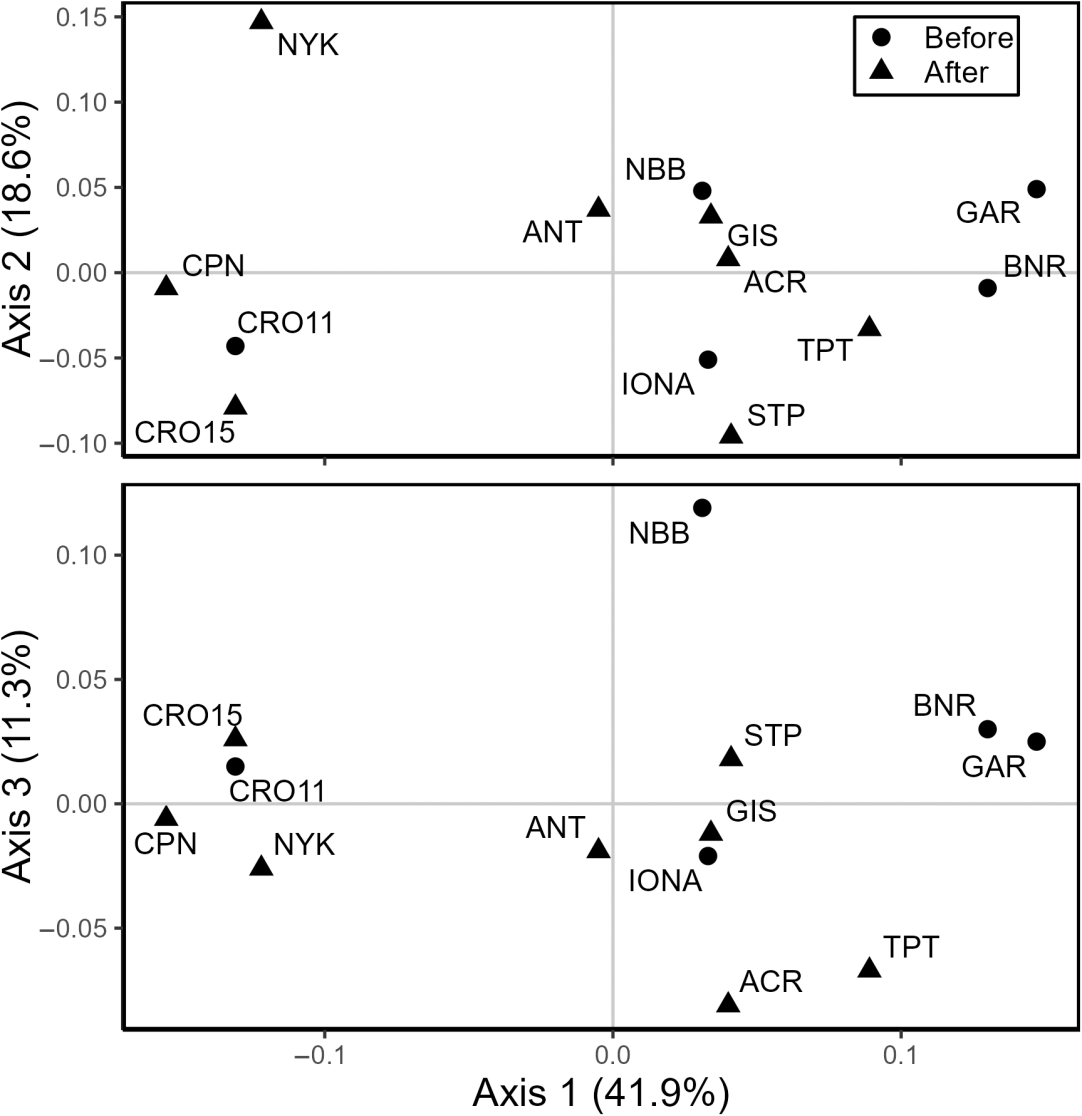
Principal coordinate analysis (PCoA) based on Nei's genetic distances among 13 *Vallisneria americana* sampling sites in the lower Hudson River Estuary. The first two axes accounted for 59.6% of explained variation (41.9% for the first axis and 18.6% for the second). The third axis accounted for an additional 11.3% of the explained variation, making a total cumulative percentage of explained variation of 71%. PCoA generally separated sites reflecting their geographical proximity rather than sampling year.

The first three PCoA axes cumulatively accounted for 71.9% of the explained variation in genetic distance, explaining 41.9%, 18.6%, and 11.3%, respectively. In PCoA space, sample sites formed three main groups that corresponded more to their geographic locations than to sampling year (Figure 7). Four downstream, higher salinity sites were separated from other sites on Axis 1 (Figure 7). NYK, the most geographically isolated site, was isolated from both Croton years (CRO11 and CRO15) and the nearby post‐storm site CPN on Axis 2 (Figure 7). The remaining sites (4 from 2011 and 6 from 2015) had no strong groupings and, although positions of sites in ordination space do not strictly reflect geographic proximity, pre‐storm sites were interspersed with post‐storm sites. Extirpation of sites BNR and GAR reduced the extent of variation along Axis 1 by 19.2% and loss of NBB reduced the extent on Axis 3 by 46.5% (Figure 7). A 90% increase in the range on Axis 2 relative to 2011 was driven by NYK.

Structure analyses indicated the most likely number of genetic groups (*K*) was two (Figure 1), and those two groups corresponded largely to location along the river gradient as seen on PCoA Axis 1 (Figure 7). Each of the two groups included pre‐ and post‐disturbance sites. A high proportion of individuals in most of the upstream sites (except GAR, with 98% assignment to the inferred upstream cluster) were admixtures of the two clusters (Figure 1). Individuals in the more downstream sites (CRO, CPN, NYK) were almost exclusively assigned to the same cluster (Figure 1).

## 4 DISCUSSION

Many sites that supported plants in 2015 had extensive and thriving beds with genotypic diversity (Table 1; Figure 3), genetic diversity (Table 1), and *N*_e_ (Table 1) similar to what we observed before the storms. We detected loss of only 4 of the 44 alleles found in 2011 and allele accumulation curves in both time periods overlapped (Figure 6). We also saw no evidence of major change in genetic structure among sample sites based on PCoA (Figure 7), *D*_est_ (Table 4), or Structure (Figure 1) analyses. These results were surprising given that impact of the 2011 storms on organisms in and around the Hudson River watershed was substantial (Hamberg et al., 2017; Michelena et al., 2019; Strayer et al., 2014). Presence of submersed aquatic vegetation at long‐term monitoring sites declined from 38% in 2011 to <3% in 2012 (Strayer et al., 2014). Absence of *V. americana* from 4 of 5 pre‐storm sample sites across a 31.2 km stretch of the river in 2015 reflected continuing impacts. Based on relationships between disturbance, demography, and genetic diversity in other aquatic species (e.g., Becheler et al., 2020; Gurgel et al., 2020; Holt et al., 2020; Manent et al., 2020), large‐scale loss of genetic diversity and changes in genetic structure seemed inevitable. Our observations appear to counter expectations and could lead us to conclude that *V. americana* had remarkable ability to maintain genetic diversity despite the extreme demographic impacts.

Yet, beds disappeared. So the storms physically removed or killed established plants and with them went their local genotypic diversity. These losses reduced the range of genetic distance on PCoA axes by ~20%–50% (Figure 7) and the extent of individual MLGs (Figures 5c and 6c). We also saw local impacts at Croton, the only sample site with plants before and after the storms. The ~20% lower GD after the storms (Table 1) was consistent with genetic effects of reduced population size in other submersed aquatic species (e.g., Diaz‐Almela et al., 2007). Even with the decline in GD, site CRO15 had among the highest genotypic diversity in either time period (Table 1). Below we discuss what these patterns tell us about short‐term mechanisms by which genetic diversity may have been maintained, implications for longer‐term ecological and evolutionary processes, and potential for resilience of *V. americana* in this river.

### 4.1 Resistance or recolonization?

In dynamic systems, both resistance to and recovery from disturbance can confer resilience over a range of temporal and spatial scales (O'Brien et al., 2018). Two MLGs found at Croton in 2011 and 2015 provide the only direct evidence of a site resisting storm damage. By contrast, ecological monitoring data and loss of 80% of our sample sites suggested that most *Vallisneria* sites could have been eliminated during the storms, requiring recovery via recolonization from relatively few refugia. If most sites were lost, we would expect post‐storm sites to have lower genotypic and genetic diversity due to founder effects, and to be more similar to one another as well as to one or a small set of founding sites. We would also expect most sites to be dominated by first‐generation migrants from those refugial sources. Those immigrants should have fewer shoots that are closer to one another than pre‐storm MLGs given the short amount of time for clonal growth between the storms and our sampling in 2015.

Our data do not meet any of these expectations. Sites had similar levels of genotypic diversity, with an average of 15 MLGs before and 14 after (Table 1). Those MLGs would have to have immigrated to post‐storm sites and propagated as many shoots (Figures 5b and 6b) over as large an extent (Figures 5c and 6c) as we found in pre‐storm sites in only three growing seasons. Shared MLGs are evidence of vegetative dispersal (Figure 5c), but the dispersal pressure needed to recolonize sites over a 24‐km stretch of the river, with each site having minimum distance of 2.6–7.8 km to the nearest neighbor, far exceeds what is known for other aquatic species (Furman et al., 2015; Kendrick et al., 2005; Nowicki et al., 2017) and would have required more propagules from more MLGs from these small, refugial sources than we found at pre‐storm sites. Required growth rates would also exceed what is known for other aquatic species, which are typically on the order of meters over decadal time scales (Kendrick et al., 2005). Structure analysis assigned all individuals into two genetic clusters with a gradient from upstream to downstream sites in the fraction of individuals assigned to each genetic cluster, suggesting some gene flow among sites (Figure 1). The number of site‐specific MLGs, PCoA structure that reflects geography (Figure 7), patterns of pairwise *D*_est_ (Table 4), and lack of significant variation attributed to year in the AMOVA‐*F*_st_ make recolonization from a limited number of refugia very unlikely and suggest the 2015 population structure was in place prior to the storms.

Thus, the combined evidence suggests that post‐storm *V. americana* beds persisted through the storms at reduced abundances that escaped detection via long‐term monitoring efforts. Small patches of *V. americana* are difficult to see in casual surveys, and if remaining shoots were not on permanent monitoring grids, they could easily have been missed. Such cryptic resistance is known to be an important but underappreciated mechanism for maintaining metapopulations in fragmented and disturbed landscapes in other species (e.g., Lamy et al., 2012; Ngeve et al., 2017).

### 4.2 Mechanisms of resistance

*Vallisneria americana* has life history, morphological, and physiological traits that could facilitate resistance. First, extensive networks of stolons and roots in clonal plants can remain intact through direct impact from hurricanes even though leaves, flowers, and fruits are sheared off and shoots are buried (Morris et al., 2004; Wilson et al., 2020). Stolon networks can have large carbohydrate reserves (Alcoverro et al., 1999, 2001) that allow persistence through low light periods. Such reserves may be the means by which *V. americana* can grow and reproduce with as low as 9%–14% of surface irradiance for extended periods (Dobberfuhl, 2007; French & Moore, 2003), survive for 3 months in complete shade (Morris et al., 2004), and tolerate burial by up to ~25 cm of sediment (Rybicki & Carter, 1986). If reserves were sufficient for turions to mature despite loss of above‐ground biomass and lack of photosynthesis in turbid post‐storm conditions, they could have overwintered and sprouted the following year.

Beyond contributing to population persistence (de Witte & Stöcklin, 2010), turion production can buffer against genetic losses because MLGs with many replicates over larger spatial extents are more likely than rare ones to persist (Bricker et al., 2018). As expected based on other aquatic species (e.g., Brzyski et al., 2018; Heidbüchel et al., 2020), most instances of the same MLG were within 200 m of one another within the same site. However, some MLGs extended across sites, including four of the five that survived the storms. Thus, extensive vegetative growth and vegetative dispersal serve as a critical mechanism for maintaining diversity even when sites are lost. Extents of 5.1–19.9 km document vegetative dispersal among sites, which has also been observed in *V. americana* in the Potomac River (Lloyd et al., 2011) and other aquatic species (Berković et al., 2018; Bricker et al., 2018). We do not know timing of dispersal except that for MLGs shared with sites that were extirpated, it had to have occurred prior to or, at the latest, during the storms.

Based on reported removal of above‐ground vegetation, we do not consider seed production from 2011 to have contributed to resistance. Considering the timing, most fruits of the current year would not have reached maturity so shearing would not have dispersed fruits with viable seeds. Seeds buried in the seedbank from previous years could be a source of recovery as they can germinate even if they are buried in <10 cm of sediment (Jarvis & Moore, 2008). However, only 1% (Jarvis & Moore, 2008) to ~10% (Lokker et al., 1997) of seeds produced make it into the seedbank and their germinability over time is not known.

Given conservation genetic principles regarding vulnerability of low‐diversity sites to stochastic losses (Ellstrand & Elam, 1993; Loveless & Hamrick, 1984) and empirical observations of genotypic diversity facilitating persistence (Hughes & Stachowicz, 2004), it is tempting to suggest genetic diversity played a role in resistance to storm damage at the higher diversity Croton site and extirpation of the low‐diversity sites. We consider it more likely that the higher diversity is a signal that Croton supported a larger or more connected population. Its relatively protected location immediately downstream of a peninsula also likely played a role (Figure 1). The extirpated sites supported smaller, sparse populations. All but NBB were immediately up‐ or downstream of the narrow, deep portion of the river where currents are especially swift. NBB, BNR, and GAR were directly exposed to downstream currents. Plants at IONA were more protected due to their location in a small cove, but that cove is at the downstream end of the narrow stretch of river that may have experienced strong eddies. We suggest that the relatively unprotected nature of these locations led to them being scoured during the 2011 floods and their small sizes before the storm could indicate chronic effects of regular scouring even in less severe floods. Thus, we suggest low diversity was a symptom of chronic disturbance rather than a driver of loss.

### 4.3 Mechanisms of regrowth

Relatively low genotypic diversity at most sites indicates that *V. americana* beds in the lower Hudson River recovered as much through asexual reproduction as through sexual, continuing the pre‐storm reproductive mode (Table 1; Figure 3). Within this regional inclination toward asexual reproduction, we saw variation among sites, with post‐storm GD values ranging from 0.14 at NYK (highly clonal) to 0.79 at ACR (more sexual reproduction). This variation suggests sites ACR, CRO15, and STP reestablished mostly from sexual reproduction as predicted by Kendrick et al. (2012), whereas others relied more on clonal growth. However, at sites ACR and CRO15 average distances among samples were higher than at other sites due to the sparseness of plants, which favors detecting more MLGs. So it is also possible that higher apparent sexual reproduction is explained by sampling distances and these sites might have shown higher clonality had we been able to find samples as close to each other as the other sites.

### 4.4 Gene flow and connectivity

Sites remaining after the storm were closer to their nearest neighbors than were sites sampled in 2011 (Figure 2a). Rather than decreasing connectivity among sampling locations, loss of sites from one stretch of the river reduced the extent of the species in the lower Hudson. This reduction decreased connectivity with the rest of the river by increasing the distance between the closest bed upstream in the freshwater reaches (at the mouth of Rondout Creek) and the salinity influenced lower Hudson from ~43 km (to NBB before) to ~74 km (to ANT after).

Pairwise *D*_est_ (Table 4) values indicate low differentiation among most of the 13 sites, but PCoA (Figure 7) denoted stronger connections among subsets of sites based on their location along the river (Figures 1 and 7). Similar pairwise *D*_est_ values among sites sampled before versus after the storm perhaps suggest no changes in gene flow patterns between both time periods, although detecting changes over short time scales is challenging. Although *D*_est_ values are likely related to the smaller spatial extent and shorter distances among post‐storm sites, these recovering beds were not more differentiated than were 2011 beds.

The one exception to connectivity among subsets of post‐storm sites is NYK, which was isolated from all other sites, both geographically (Figure 1) and in PCoA space (Figure 7). In addition to geographic distance, NYK may be isolated by soft barriers induced by higher salinity. Lloyd et al. (2011) found a similar apparent break in connectivity at *V. americana* sites associated with higher salinity in the Chesapeake Bay. The distance and *D*_est_ values for NYK are based on the 5 MLGs found at that site. The small number of MLGs reflects the relative geographic isolation of this site and the PCoA results show potential signs of genetic drift due to that isolation. Effects of ongoing drift are further implicated by the strongly skewed abundances of the five MLGs, yielding the lowest GD_Simpson_ value of any site (Table 1). Strong selection under high salinity is also a possibility, but we cannot test for selection with microsatellite markers.

### 4.5 Conclusions and management implications

The observed demographic recovery at many sites, lack of dramatic loss of genotypic and genetic diversity, and absence of inbreeding leave us optimistic for the future of *V. americana* in the lower Hudson River Estuary. Additional optimism comes from the expectation that species living in dynamic ecosystems survive continuously changing conditions and frequent disturbances. Some researchers have even suggested evidence for chronic disturbance selecting for genotypes that are more broadly tolerant of harsher conditions and thus resistant to acute disturbances (Connolly et al., 2018). Even without major disturbance, annual variation in water clarity (i.e., light availability) yields high variability in the number, size, and location of *V. americana* beds in the Hudson (Findlay et al., 2014) and elsewhere (Lefcheck et al., 2018; Orth et al., 2017; Rybicki & Landwehr, 2007), which may facilitate adaptation to dynamic conditions.

We are cautious, however, because a less positive explanation for the apparent lack of decrease in genetic diversity is that limited pre‐storm data, or extremely low pre‐storm levels of diversity precluded detecting declines. If losses at 2015 sites, for which we have no pre‐storm data, were of similar magnitude to losses at Croton, their similarity in genotypic and genetic diversity to levels found at extirpated sites may signal a downward trend of diversity and increased vulnerability. When compared to other western Atlantic estuaries (Lloyd et al., 2011, 2016; Marsden et al., 2022), lower Hudson River *Vallisneria* beds were genetically depauperate even before the storms. Signatures of recent genetic bottlenecks in 6 out of 13 sites, *N*_e_ values that were far lower than is considered viable (Franklin, 1980), and low GD at most sites (Table 1) may reflect recurring disturbance even before the 2011 storms and cause concern.

Given these diversity levels reflect conditions in the river that pre‐date the storms, just how much concern is warranted is not clear. It is even possible that low diversity in the Hudson is a consequence of founder events during colonization after post‐Pleistocene deglaciation. Pollen cores in nearby lakes indicate suitable conditions have been in place for other species at approximately 12.5 ka (Peteet et al., 2009). Low diversity (Diekmann & Serrão, 2012; Dorken & Eckert, 2001) and predominantly asexual reproduction are common near the leading range edge for many species (e.g. Eckert et al., 2008). However, the 2011 storms occurred on a backdrop of chronic anthropogenic effects that had already reduced the species from historic levels in recent times (Findlay et al., 2014; Nieder et al., 2009; Strayer et al., 2014). If the low levels of diversity are due to these more recent population reductions that favor clonality, concern would be higher than if they are long‐term patterns the species has survived.

Clonality, which causes the observed low GD, is a double‐edged sword. It contributed greatly to survival and maintenance of genetic diversity through extreme disturbance. At the same time, relatively few MLGs represent over 60% of the shoots sampled, which is reflected in extreme unevenness among MLGs (Figure 3). Low genotypic diversity can be self‐reinforcing because a larger proportion of shoots will be from asexual reproduction and the same MLGs will become more extensive. As they expand, the sexes can become isolated (Barrett, 2015; Doust & Laporte, 1991; Honnay & Bossuyt, 2005; Lokker et al., 1994, 1997), further reducing opportunities for sexual reproduction and generating increasingly large MLGs (Eriksson, 1989). The effects of dominance by extensive clones can be amplified to other sites because large numbers of ramets and associated turions provide many opportunities for vegetative dispersal. Ability of such processes to yield extensive clonal dominance after colonization has been demonstrated through modeling (Rafajlović et al., 2017), and mechanisms for vegetative dispersal have been determined experimentally (Lai et al., 2018).

Hindrances to sexual reproduction have long‐term consequences for future resilience because there is little opportunity for natural selection when numbers of individuals and amounts of genetic variation are low. Such impediments can contribute to the self‐reinforcing cycle that further limit opportunities for sexual reproduction. The long‐term risk posed by low genotypic diversity depends on whether the few remaining dominant MLGs have sufficient breadth of tolerance to acclimate to changing environmental conditions. If so, there is little concern for resilience even if MLGs are widespread and dominant across multiple sites. If MLGs have narrow tolerances and differ in their optimal environment, resilience would require a diverse array of MLGs and dominance by a few may compromise future acclimation and adaptation (see Pazzaglia et al., 2021). Further, reliance on acclimation through phenotypic plasticity of clones may not provide enough insurance against the collapse of large beds or even the entire system, if environmental variability exceeds environmental tolerances of the few remaining resident genotypes, or if new stressors are introduced to the system. As such, better understanding of these tolerances is key to assessing the need to augment diversity.

Although the genetic consequences appear limited, the obvious ecological consequence of the storms is absence of *V. americana* beds from a 31.2 km stretch of the river between IONA and NBB. On the one hand, the absences could be viewed as a shift in specific locations of the species within a regionally stable mosaic of sites. Alternatively, they could represent long‐term degradation and a regime shift to dominance by ephemeral species or bare sediment (Kendrick et al., 2019; O'Brien et al., 2018). Sites are within distances of past dispersal as indicated by PCoA clusters, *D*_est_ values, and shared MLGs. Dispersal is enhanced by the tidal nature of the river that enables propagules to go up‐ and downstream. But still, the probability of recolonization in any year is low and could take longer to return ecological function fulfilled by *V. americana* than managers are willing to accept. A reasonable management action would be to restore the plant in previously occupied locations to jumpstart the recolonization and expansion. Appropriate sourcing of propagules for augmentation efforts is crucial to avoid inbreeding and outbreeding depression (Marsden et al., 2013) at planted sites. To maintain existing genetic structure, we recommend choosing propagules from the closest sites. We also recommend choosing propagules to increase potential for sexual reproduction in restored sites, which requires introducing male and female MLGs.

Ongoing monitoring using permanent grid points and periodic mapping from aerial photography provide critical information on dynamics of *V. americana* beds in the river. Continuing to monitor extirpated sites will provide critical data on recolonization over time. Our results show, however, that the fixed points underestimated the extent of persistence through the storms because individual shoots and small patches did not coincide with monitoring points. Field counts also do not give insight into how many of the observed shoots are the same clonal individual. Complementing demographic data periodically with genetic monitoring would provide insight into changing numbers of MLGs, genetic diversity levels, and mode of reproduction over time as beds continue to establish and expand. Such data are needed to determine whether diversity increases over time as populations recover from acute disturbance or diversity continues to decline through genetic drift, yielding increased reliance on clonal growth. Such data are critical for assessing risk and strategizing management efforts that can better understand effects of disturbance and ensure resilience.

## AUTHOR CONTRIBUTIONS

**Magdalene N. Ngeve:** Conceptualization (supporting); data curation (supporting); formal analysis (supporting); methodology (supporting); visualization (supporting); writing – original draft (lead); writing – review and editing (supporting). **Katharina A. M. Engelhardt:** Conceptualization (equal); funding acquisition (equal); writing – original draft (supporting); writing – review and editing (supporting). **Michelle Gray:** Data curation (supporting); methodology (supporting); writing – review and editing (supporting). **Maile C. Neel:** Conceptualization (equal); data curation (lead); investigation (lead); methodology (lead); project administration (lead); resources (lead); supervision (lead); visualization (lead); writing – original draft (equal); writing – review and editing (lead).

## CONFLICT OF INTEREST STATEMENT

The authors declare we have no competing interests.

## PERMISSION TO REPRODUCE MATERIALS FROM OTHER SOURCES

None.

## Data Availability

Microsatellite genotype data and additional analysis files are available on Dryad at https://doi.org/10.5061/dryad.fn2z34v0s.
